# Epidemiology of Neuromyelitis Optica Spectrum Disorder and Its Prevalence and Incidence Worldwide

**DOI:** 10.3389/fneur.2020.00501

**Published:** 2020-06-26

**Authors:** Jyh Yung Hor, Nasrin Asgari, Ichiro Nakashima, Simon A. Broadley, M. Isabel Leite, Najib Kissani, Anu Jacob, Romain Marignier, Brian G. Weinshenker, Friedemann Paul, Sean J. Pittock, Jacqueline Palace, Dean M. Wingerchuk, Jacinta M. Behne, Michael R. Yeaman, Kazuo Fujihara

**Affiliations:** ^1^Department of Neurology, Penang General Hospital, Penang, Malaysia; ^2^Department of Neurology, Institute of Molecular Medicine, University of Southern Denmark, Odense, Denmark; ^3^Department of Neurology, Tohoku Medical and Pharmaceutical University, Sendai, Japan; ^4^Menzies Health Institute Queensland, Griffith University, Southport, QLD, Australia; ^5^Department of Neurology, Gold Coast University Hospital, Southport, QLD, Australia; ^6^Nuffield Department of Clinical Neurosciences, University of Oxford, Oxford, United Kingdom; ^7^Neurology Department and Neuroscience Research Laboratory of Marrakech Medical School, University Hospital Mohammed VI, Marrakech, Morocco; ^8^Walton Centre NHS Foundation Trust, Liverpool, United Kingdom; ^9^Cleveland Clinic Abu Dhabi, Abu Dhabi, United Arab Emirates; ^10^Service de Neurologie, Sclérose en Plaques, Pathologies de la Myéline et Neuro-inflammation, and Centre de Référence des Maladies Inflammatoires Rares du Cerveau et de la Moelle, Hôpital Neurologique Pierre Wertheimer, Hospices Civils de Lyon, Lyon, France; ^11^Department of Neurology, Mayo Clinic, Rochester, MN, United States; ^12^NeuroCure Clinical Research Center, Charité—Universitätsmedizin Berlin, Corporate Member of Freie Universität Berlin, Humboldt-Universität zu Berlin, and Berlin Institute of Health, and Experimental and Clinical Research Center, Max Delbrueck Center for Molecular Medicine and Charité—Universitätsmedizin Berlin, Berlin, Germany; ^13^Department of Neurology, Mayo Clinic, Scottsdale, AZ, United States; ^14^The Guthy-Jackson Charitable Foundation, Beverly Hills, CA, United States; ^15^Divisions of Molecular Medicine and Infectious Diseases, David Geffen School of Medicine at UCLA, Los Angeles and Harbor-UCLA Medical Center, Lundquist Institute for Biomedical Innovation at Harbor-UCLA Medical Center, Torrance, CA, United States; ^16^Department of Multiple Sclerosis Therapeutics, Fukushima Medical University School of Medicine, and Multiple Sclerosis and Neuromyelitis Optica Center, Southern TOHOKU Research Institute for Neuroscience, Koriyama, Japan

**Keywords:** neuromyelitis optica spectrum disorder, NMOSD, AQP4, MOG, prevalence, incidence, population study, epidemiology

## Abstract

Neuromyelitis optica spectrum disorder (NMOSD) is an uncommon inflammatory disease of the central nervous system, manifesting clinically as optic neuritis, myelitis, and certain brain and brainstem syndromes. Cases clinically diagnosed as NMOSD may include aquaporin 4 (AQP4)-antibody-seropositive autoimmune astrocytopathic disease, myelin oligodendrocyte glycoprotein (MOG)-antibody-seropositive inflammatory demyelinating disease, and double-seronegative disease. AQP4-antibody disease has a high female-to-male ratio (up to 9:1), and its mean age at onset of ~40 years is later than that seen in multiple sclerosis. For MOG-antibody disease, its gender ratio is closer to 1:1, and it is more common in children than in adults. Its clinical phenotypes differ but overlap with those of AQP4-antibody disease and include acute disseminated encephalomyelitis, brainstem and cerebral cortical encephalitis, as well as optic neuritis and myelitis. Double-seronegative disease requires further research and clarification. Population-based studies over the past two decades report the prevalence and incidence of NMOSD in different populations worldwide. One relevant finding is the varying prevalence observed in different racial groups. Consistently, the prevalence of NMOSD among Whites is ~1/100,000 population, with an annual incidence of <1/million population. Among East Asians, the prevalence is higher, at ~3.5/100,000 population, while the prevalence in Blacks may be up to 10/100,000 population. For MOG-antibody disease, hospital-based studies largely do not observe any significant racial preponderance so far. This disorder comprises a significant proportion of NMOSD cases that are AQP4-antibody-seronegative. A recent Dutch nationwide study reported the annual incidence of MOG-antibody disease as 1.6/million population (adult: 1.3/million, children: 3.1/million). Clinical and radiological differences between AQP4-antibody and MOG-antibody associated diseases have led to interest in the revisions of NMOSD definition and expanded stratification based on detection of a specific autoantibody biomarker. More population-based studies in different geographical regions and racial groups will be useful to further inform the prevalence and incidence of NMOSD and their antibody-specific subgroups. Accessibility to AQP4-antibody and MOG-antibody testing, which is limited in many centers, is a challenge to overcome. Environmental and genetic studies will be useful accompaniments to identify other potential pathogenetic factors and specific biomarkers in NMOSD.

## Introduction

Neuromyelitis optica spectrum disorder (NMOSD) is an uncommon inflammatory disease of the central nervous system, with clinical features of optic neuritis, myelitis, and certain brain, and brainstem syndromes. Although it had long been debated whether NMOSD is a severe variant of multiple sclerosis (MS), the discovery of NMOSD-specific aquaporin 4 (AQP4) antibody, and the subsequent clinical, immunological, and pathological data have established that NMOSD is indeed a distinct entity (1–3). Currently, cases clinically diagnosed as NMOSD may include AQP4-antibody-seropositive autoimmune astrocytopathic disease, myelin oligodendrocyte glycoprotein (MOG)-antibody-seropositive inflammatory demyelinating disease, and double-seronegative disease (4).

AQP4-antibody-seropositive NMOSD has a high female-to-male ratio (up to 9:1) (5), and its mean age at onset is around 40 years (6, 7), older than in MS. Pathologically, it is primarily an astrocytopathic disease rather than a demyelinating disease (3, 8). For MOG-antibody disease, the sex ratio is close to 1:1, and it is more common in children than in adults (9, 10). Its clinical manifestations overlap with those of AQP4-antibody disease but there are differences about which there is emerging consensus. Besides optic neuritis and myelitis, its clinical phenotypes also include acute or multiphasic disseminated encephalomyelitis (ADEM/MDEM), brainstem and cerebral cortical encephalitis, and cranial nerve involvement (11–14). Double (AQP4- and MOG-antibodies)-seronegative disease is enigmatic at present and requires further clinical and laboratory research for specific classification.

There have been several editions of the diagnostic criteria for NMOSD since 1999 (15, 16), with the latest being the 2015 International Panel on NMO Diagnosis (IPND) criteria (17). In the meantime, laboratory assays for AQP4 antibody and MOG antibody have also improved over time, with increased sensitivity and specificity (18, 19). These factors have contributed to the improvement in the accuracy of the diagnosis of NMOSD cases.

In this article, we review current data on the worldwide epidemiology of NMOSD, specifically on the population-based studies of NMOSD to determine its prevalence and incidence among different populations and racial groups. We emphasize that the field of NMOSD is undergoing a rapid evolution, making epidemiological estimates tentative. Additionally, different levels of diagnostic rigor to exclude NMOSD mimics and access to medical care in study populations can bias the epidemiological survey results in the disease, which makes the interpretation and comparison of the findings in and across the studies difficult. Nonetheless, the best known of current knowledge is being presented.

### Search Strategy and Selection Criteria

The PubMed database was searched for population-based studies on NMOSD with prevalence data, from 1st January 2000 till 11th March 2020. A combination of the following search terms was used: “neuromyelitis optica,” “NMO,” “NMOSD,” “aquaporin 4,” “AQP4,” “myelin oligodendrocyte glycoprotein,” “MOG,” “optico-spinal multiple sclerosis,” “OSMS,” “idiopathic inflammatory demyelinating disease,” “IIDD,” “epidemiology,” “prevalence,” “population,” and “demographic.” The reference lists in published articles on NMOSD were also queried to identify further studies. Additionally, recent conference proceedings of major neurology and MS congresses, including the European Committee for Treatment and Research in Multiple Sclerosis (ECTRIMS) and the Pan-Asian Committee for Treatment and Research in Multiple Sclerosis (PACTRIMS), were searched for relevant abstracts where the full studies are not yet published. Population-based studies with information on the prevalence of NMOSD in English language were reviewed. The final list of publications was selected on the basis of relevance to the topic.

### Prevalence of NMOSD

The prevalence range of NMOSD is ~0.5–4/100,000, and may be up to 10/100,000 in certain racial groups. Nevertheless, this prevalence range is rather small relative to that of MS, which ranges from 1–2/100,000 in the equatorial region, to 150–200/100,000 in Canada and northern part of Europe (20, 21).

Over the past two decades, population-based studies of NMOSD have provided important insights into its prevalence. The earliest population-based studies were conducted in French West Indies (Martinique) (22, 23), Cuba (24), Denmark (25), and Tokachi Province on Hokkaido Island in Japan (26). Interestingly, the majority of these early studies were conducted on island populations, which facilitate population-based studies by providing well-delimited boundaries of the study area. Two of the studies (Martinique and Hokkaido) (22, 23, 26) have since been updated by the original groups of researchers.

**Figure 1:**
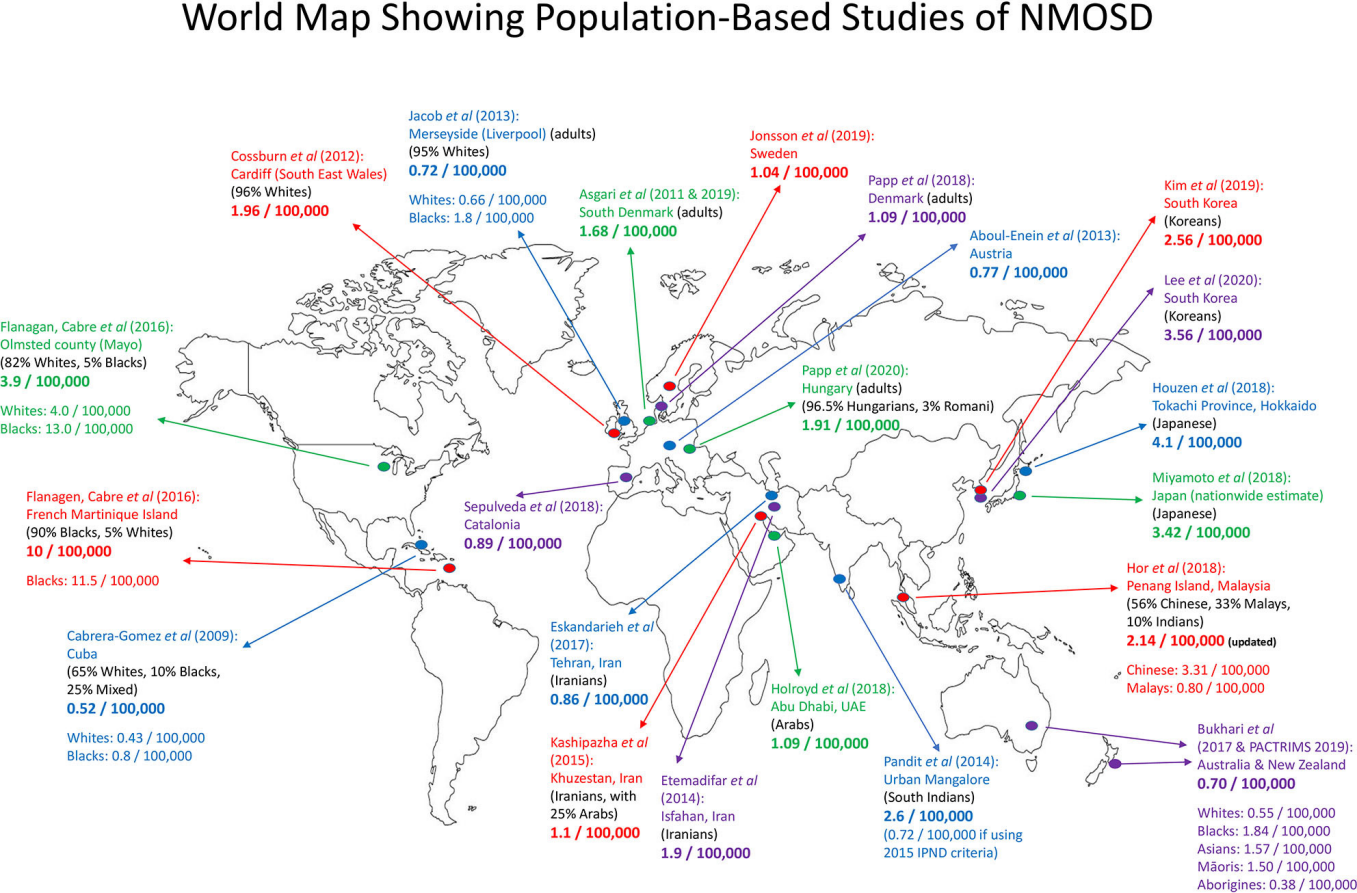
Map showing population-based prevalence studies of NMOSD around the world. There were eight studies in Europe, 10 in Asia, one in Oceania, and two in the Americas (one in Cuba and one joint study in the USA and Martinique Island). Numbers given were prevalence per 100,000 population. In certain studies, the prevalence according to racial groups was given. Adults, only adult population was studied.

Since 2017, several new population-based studies were published, expanding knowledge of NMOSD in diverse populations around the world. Inter-racial variation in prevalence, as summarized in Table 1, is notable and consistent across geographical regions. More recently, the Australia/New Zealand group has also re-analyzed the data from their 2017 study (47) to provide further information with regards to the prevalence among different racial groups in their large continent (46). Figure 1 is a map showing population-based prevalence studies of NMOSD around the world.

**Table 1:** Population-based prevalence and incidence studies of NMOSD.

**Population-based study**	**Geographical location**	**Prevalence of NMOSD (per 100,000 population), as according to racial groups**				**Incidence (per million population)**	**AQP4-ab testing methods**	**AQP4-ab positivity**	**Female-to-male ratio**
		**Whites/Caucasians**	**Blacks**	**East Asians**	**Other Asians/Other Races**				
Cabrera-Gomez et al. (2009) (24)	Cuba	0.43	0.80			0.53	Not tested	Not tested	7.3:1
Asgari et al. (2011) (25) (re-analyzed 2019) (27)	South Denmark	1.68^*^				1.5	CBA	62%	5.3:1
Cossburn et al. (2012) (28)	South East Wales	1.96				NR	NR	71%	6:1
Jacob et al. (2013) (29)	Merseyside, England	0.66^*^	1.8^*^			0.8	Oxford CBA	88%	3:1
Aboul-Enein et al. (2013) (30)	Austria	0.77				0.54	Innsbruck CBA	100%	7:1
Pandit and Kundapur (2014) (31)	Mangalore, India				South Indians: 2.6 (0.72 if using 2015 IPND criteria)	NR	NR	27%	1.2:1
Etemadifar et al. (2014) (32)	Isfahan, Iran	1.9				NR	NR	66%	2.3:1
Kashipazha et al. (2015) (33)	Khuzestan, Iran	1.1				NR	NR	54%	7.5:1
Flanagan et al. (2016) (34)	Olmsted county, USA	4.0	13.0			0.7	Mayo CBA	83%	5:1
	French Martinique Island	6.1 (single case, AQP4-ab negative)	11.5			7.3	Mayo CBA	79%	8.8:1
van Pelt et al. (2016) (35)	Netherlands	—				0.9	CBA	NA	4.9:1
Houzen et al. (2017) (36)	Tokachi, Hokkaido, Japan			Japanese: 4.1		NR	Sendai CBA	79%	6:1
Eskandarieh et al. (2017) (37)	Tehran, Iran	0.86				NR	ELISA	47%	5.1:1
Sepúlveda et al. (2018) (38)	Catalonia	0.89				0.63	Mainly CBA (96%)	73%	3.1:1
Hor et al. (2018) (39)	Penang Island, Malaysia			Chinese: 3.31	Malays: 0.80 (revised)	NR	Euroimmun CBA	100%	14:1
Miyamoto et al. (2018) (40)	Japan (nationwide estimate)			Japanese: 3.42		NR	NA	NA	6.4:1
Holroyd et al. (2018) (41)	Abu Dhabi, UAE				Arabs: 1.09	1.16	NR	83%	All females
Papp et al. (2018) (42)	Denmark	1.09^*^				0.70	Various, incl. CBA	70%	4.5:1
Jonsson et al. (2019) (43)	Sweden	1.04				0.79	Immunoblot and CBA	NR	2.8:1
Kim et al. (2019) (44)	South Korea			Koreans: 2.56		7.3	CBA	NA	2.37:1
Papp et al. (2020) (45)	Hungary	Hungarians: 1.91^*^				1.32	CBA	83%	8.8:1
Bukhari et al.	Australia and	0.55	1.84	Asians: 1.57		0.37	IF tissue	>90%	6:1
(PACTRIMS	New Zealand				Māoris: 1.50		assay,		
2019) (46) (updated from 2017 study) (47)					Australian Aborigines: 0.38		some also ELISA and CBAs		
Lee et al. (2020) (48)	South Korea			Koreans: 3.56		4.1–6.5	NA	NA	4.7:1

* *Only consider adult population. (As AQP4-antibody-positive NMOSD is rare in children, thus, if full population is considered, the prevalence will be slightly lower)*.

*NMOSD, neuromyelitis optica spectrum disorder; AQP4-ab, aquaporin 4-antibody; CBA, cell-based assay; IF, immunofluorescence; NA, not applicable; NR, not reported*.

#### East Asians

East Asians (Japanese, Chinese, and Koreans) appear to have a higher prevalence of NMOSD (around 3.5/100,000) as compared to Whites and other Asian racial groups. The study in Hokkaido, Japan recorded a prevalence of 4.1/100,000 (36), while the Japanese nationwide survey estimated a prevalence of 3.42/100,000 (40). Meanwhile, a study conducted in the multi-racial population in Penang Island, Malaysia showed that the prevalence among Chinese was 3.31/100,000 (39). These results were in line with the genetic studies that showed that Japanese and Chinese share the same HLA risk genes for NMOSD, namely, HLA-DPB1^*^05:01 and HLA-DRB1^*^16:02 (49–51). In a very recent study from South Korea, by using a nationwide health insurance research dataset, it was calculated that the prevalence among Koreans was 3.56/100,000 in 2017 (48). More studies, especially from China, Taiwan, and Hong Kong will be useful to further inform the prevalence of NMOSD among the East Asians.

#### Blacks

In 1971, a study conducted in a single hospital in the sub-Saharan African city of Ibadan (Nigeria) reported 95 cases of NMO, 22 cases of acute transverse myelitis, 11 cases of bilateral retrobulbar neuritis, and only two cases of MS over 12 years of hospital admissions (1957–1969) (52). During the same period, there were nine cases of non-Nigerians with MS (in eight Europeans and one Indian). It estimated that NMO cases made up 0.43/1000 (or 430/100,000) of the hospital population.

Population-based studies over the past two decades showed that Blacks also have a higher NMOSD prevalence than Whites. A study conducted in Liverpool, UK reported a prevalence rate of 1.8/100,000 among Blacks (29). The Australia/New Zealand study estimated a prevalence rate of 1.84/100,000 in those with African ancestry (46). The study conducted in the French Martinique Island in the Caribbean reported a very high prevalence of 11.5/100,000 among its Black population (34), and this was the highest prevalence reported so far. In population-based studies, within the same localities, prevalence among Blacks is always higher than in Whites, as seen in Cuba (24), Liverpool (UK) (29), Olmsted county (USA) (34), Martinique Island (34), and Australia/New Zealand (46).

As Blacks are genetically diverse, more data from different geographical regions are needed, and especially those from the African continent. Although no population-based studies of NMOSD have been published from Africa, recently there have been reports of NMOSD cases from various African countries that are to be compiled and reported elsewhere.

#### Whites/Caucasians

In recent nationwide and region-wide studies, the prevalence of NMOSD among Whites has consistently been ~1/100,000. The prevalence was 0.55/100,000 in Australia and New Zealand (46, 47), 0.89/100,000 in Catalonia (38), 1.09/100,000 in Denmark (42), and 1.04/100,000 in Sweden (43). Also recently, a re-analysis of the data of an earlier study from South Denmark has reported the prevalence of AQP4-antibody-positive NMOSD as 1.68/100,000, and the prevalence of the total clinical phenotype including AQP4-antibody-negative and MOG-antibody-positive subsets was 4.4/100,000 (25, 27).

Interestingly, the prevalence among Hungarians was slightly higher, at 1.91/100,000 (45). This has brought up the notion of whether there are some admixtures of Asian genes (from North East Asia) among the Hungarians (53). Furthermore, there is scarcity of prevalence data from Central Asia, and such data from this region will be informative.

### Other Asians

#### South Indians

If the 2015 IPND criteria were applied, the prevalence among South Indians in Mangalore was 0.72/100,000 (31). No cases were found among the 10% South Indian population in Penang Island, Malaysia (39), suggesting a low prevalence.

#### Austronesian Peoples

The Austronesian peoples reside in the Philippines, Malaysia, Indonesia, the Pacific Islands (Polynesia, Micronesia, and Hawaii), down to New Zealand, and also to the west in Madagascar. The study conducted in the multi-racial Penang Island, Malaysia (39) found that the prevalence of an Austronesian group, the Malays, was ~0.80/100,000 (this was revised from 0.43/100,000 as reported earlier, after a new case was diagnosed). The prevalence data from another Austronesian group was available recently, namely, the Māoris in New Zealand, with an estimated prevalence of 1.50/100,000 (46). Nevertheless, in the same study, no cases of NMOSD were found among the ~295,000 Pacific Islanders (Pasifika) (46). More data from other Austronesian groups in other localities will be useful to clarify this.

#### Arabs

A study from Abu Dhabi, United Arab Emirates reported six cases of NMOSD among its citizens, consistent with a prevalence of 1.09/100,000 (AQP4-antibody seropositivity: 83%, all six cases were females) (41). If only adult citizens aged ≥20 years were considered (a total of five cases), the prevalence is higher at 1.76/100,000. Data on Arabs in other regions of Middle East and North Africa will be very informative.

#### Australian Aborigines

The Australian Aborigines are one of the oldest populations in the world, with their ancestors having migrated to Australia around 50,000 years ago. There is evidence of some admixture of Denisovan genes in the Aborigines (Denisovans are an extinct species or subspecies of humans of the genus *Homo*). It is interesting to note that MS rarely exists in the Aborigines (54, 55). Recent data showed that NMOSD is also rare among the Aborigines, with a prevalence of 0.38/100,000 (46). However, the paper cautioned whether inequality in health care access may lead to this low figure.

#### Native Americans

MS is less common among Native Americans than in Whites in North America. Prior to AQP4-antibody discovery, a study conducted among the Native Canadians in Manitoba (56) found seven cases of “MS,” of which five cases were of NMO phenotypes, while the other two had brainstem involvement. Autopsy of one patient showed eosinophil infiltration in the cervical cord lesion, and retrospectively, this pathological finding suggests that this case was likely to be NMOSD. Genetically, Native Americans may be more closely related to early East Asians, and thus they may also have a higher prevalence than Whites. A re-look at these native populations will be helpful to confirm the results, though may be practically difficult.

#### Latin America

After the arrival of Europeans in the 1500's, the indigenous populations of Latin American had dwindled rapidly. Today, along with the indigenous peoples, there is a large proportion of Whites, Blacks, and mixed races in Latin America.

In an earlier study from a tertiary hospital in Mexico City (57), using 1999 Wingerchuk criteria, a total of 34 cases of NMO were identified, with all patients being Mestizos (mixed race). By calculating the ratio of MS and NMO in the hospital, and by using the estimated MS prevalence in the country at that time, it was extrapolated that the prevalence of NMO among Mexican Mestizos was around 1.3/100,000. With the availability of AQP4-antibody assays, and the newer diagnostic criteria that include NMOSD cases, this prevalence rate is likely to be higher.

From the preliminary findings of a recent study involving seven general hospitals in Venezuela presented at a conference (58), it was estimated that the prevalence of NMOSD in Venezuela was 2.2/100,000, with a female-to-male ratio of 4:1, and again Mestizos formed the majority of those patients.

Studies from other representative populations will be useful to further inform the prevalence of NMOSD in Latin America.

#### North Africa

The populations of North Africa consist mainly of Amazighs (Berbers) and Arabs. As in Whites/Caucasian populations, there appear to be much higher number of MS than NMOSD cases in North Africa (59, 60). There have been no population-based studies on NMOSD in North Africa so far. There is only one population-based study on Arabs in the Middle East (Abu Dhabi) (41), and the prevalence data among Amazighs are awaited.

### Incidence of NMOSD

Table 1 summarizes the incidence reported in the available population-based studies. Among Whites, the annual incidence of NMOSD is generally reported to be around 0.5–0.8/million (30, 38, 42, 43). In populations with a higher prevalence, the incidence is also higher. For instance, Blacks in Martinique have a high prevalence of 11.5/100,000, and its incidence was also reported to be high, at 7.3/million (34). Recently, the data from South Korea also showed a high incidence, ranging from 4.1 to 7.3/million for the period 2013–2017 (44, 48). Other populations with a prevalence higher than 1/100,000 also reported an incidence higher than 1/million [for example, 1.16/million in Arabs (41), and 1.32/million in Hungarians (45)].

A limitation regarding incidence calculation is that, if a new antibody test becomes available in the study region, or when there is increased awareness among clinicians, then the number of newly diagnosed cases in that particular year will be higher, leading to a higher incidence rate, even though the disease could have started many years earlier in some cases. Nevertheless, if researchers are able to calculate the incidence rates over the past few years (e.g., past 5 years) and average them, it is likely to be more accurate.

For pediatric NMOSD, there were two recent nationwide/region-wide studies that reported on its incidence. In the Danish study, the incidence of pediatric NMOSD was calculated as 0.31/million (61). In the Taiwanese study using the national health insurance research database, over the period from 2011 to 2015, the average annual incidence was reported as 1.1/million (62). Again, this higher incidence in Taiwan as compared to Denmark is not surprising as NMOSD is more prevalent among East Asians than Whites.

### Age and Racial Differences in the Clinical Features and Severity of NMOSD

Some studies have analyzed how the clinical features and disability are affected by onset age and racial differences. Patients with young-onset NMOSD were more likely to have optic neuritis as onset attack, while older-onset patients often developed myelitis as the initial presentation (63). Furthermore, young-onset patients with optic neuritis were more likely to develop not only recurrent optic neuritis but also higher likelihood of developing blindness, as compared to older-onset patients with optic neuritis (63, 64). Conversely, older-onset patients with myelitis often had poor recovery, while most young-onset patients with myelitis recovered well without permanent motor disability (63, 64).

There also appears to be some differences in the clinical features of NMOSD among different races. Blacks and Asians tended to have lower mean ages at onset than Whites (Blacks: around 28–33 years, Asians: 35–40 years, vs. Whites: 44 years) (63, 65). Black and Asian patients were more likely to have brain and brainstem attacks and abnormalities on brain MRI as compared to Whites (64, 65). Overall, the risk of relapse was lowest in Japanese than in Whites and Blacks (63, 64).

Blacks were found to have a greater likelihood of developing visual disability with time than Whites and Japanese (63, 64). On the other hand, Whites had a higher probability of developing severe motor disability or wheelchair dependence as compared to Japanese (63). Severe attacks were more frequent in Blacks than in Asians and Whites, and therefore Blacks were at a higher risk of severe disability in the early course of the disease (65). In a study from the USA, patients with African ancestry were also found to have a higher mortality rate (15.4%) as compared to the overall mortality rate (7.0%) (66). Nonetheless, while race affected the clinical phenotype, age at onset, and severity of attacks, the overall outcomes were mostly dependent on early and effective immunosuppressive treatment (65).

### MOG-Antibody-Associated Disease: Prevalence and Incidence

After the discovery of the AQP4 antibody, a majority of NMO cases have been found positive for this antibody. Nevertheless, there is still a proportion of cases with an NMO phenotype that are persistently tested negative for AQP4 antibody, despite using the most sensitive cell-based assays available. It was later realized that some of these AQP4-antibody-negative NMOSD cases were in fact seropositive for MOG antibody. This so-called MOG-antibody-associated disease consists of a significant proportion of NMOSD cases that are AQP4-antibody seronegative, ranging from 7 to 42% (7, 67–70).

Interestingly, for MOG-antibody-associated disease, besides NMO phenotype, optic neuritis, and myelitis, some of these MOG-antibody-positive cases also have clinical phenotypes beyond the current NMOSD spectrum, such as ADEM/MDEM-like presentation (71), cerebral cortical encephalitis (12), and cranial nerve involvement (14). Pathologically, MOG-antibody-associated disease is a type of demyelinating disease, as opposed to astrocytopathic disease seen in AQP4-antibody-positive NMOSD (72, 73).

A recent Dutch nationwide study reported the incidence of MOG-antibody-associated disease as 1.6/million, with 1.3/million in adults, and a higher incidence of 3.1/million in children (74). It should be noted that this incidence rate of 1.6/million is higher than the incidence rate of 0.5–0.8/million in NMOSD (mostly AQP4-antibody-positive) among Whites.

So far, hospital-based studies largely did not observe any significant racial preponderance for MOG-antibody-associated disease. For instance, in the UK cohort, the racial breakdown was as expected in the general population (9). Nevertheless, from the annual report of the Oxford NMO Service, there were 145 patients with AQP4-antibody-positive NMOSD, 111 patients with MOG-antibody disease, and 28 patients who were double-seronegative. The proportion of MOG-antibody disease within the NMOSD spectrum was rather significant (75). Additionally, a study from Mayo Clinic on AQP4- and MOG-antibody testing for 15,598 patients showed higher positivity rate for MOG antibody (1291 patients, 8.3%) than for AQP4 antibody (387 patients, 2.3%). Of the adults, 6.5% were MOG-antibody positive vs. 2.6% for AQP4 antibody, while in children, 21.1% were positive for MOG antibody as compared to 1.9% for AQP4 antibody (76). Similarly, one study in Sri Lanka, in collaboration with the Mayo Clinic, also reported more MOG-antibody-positive cases (126 patients) than AQP4-antibody-positive cases (36 patients) (77). On the other hand, MOG-antibody-associated disease was relatively uncommon in the non-Caucasian population in Rio de Janeiro (Brazil) (70).

The preliminary findings of a population-based prevalence study of MOG-antibody-associated disease, jointly conducted at Olmsted county (USA) and Martinique Island, were recently presented at a conference (ECTRIMS 2019) (78). In Olmsted county, the prevalence was calculated to be 3.42/100,000, with an incidence of 2.39/million, while at Martinique, the prevalence was 1.6/100,000, with an incidence of 1.12/million.

In the Catalonia NMOSD prevalence study, 12% of cases were MOG-antibody-positive (38). However, the cases in this study were required to strictly fulfill the 2015 IPND criteria, and thus only those with an NMO phenotype were analyzed (The prevalence of MOG-antibody-positive NMO was calculated to be 0.11/100,000.). Needless to say, if MOG-antibody-positive cases with optic neuritis alone or myelitis alone and those with ADEM-like presentation are included, the prevalence of MOG-antibody disease is likely to be higher.

More data from different geographical areas are clearly in need to further inform about the prevalence and incidence of MOG-antibody-associated disease.

Some demographic and epidemiological data and clinical features of AQP4-antibody-positive NMOSD and MOG-antibody-associated disease in comparison with MS are shown in Table 2.

**Table 2:** Epidemiological and clinical comparison between AQP4-antibody-seropositive NMOSD, MOG-antibody disease, and MS.

	**AQP4-antibody disease**	**MOG-antibody disease**	**MS**
Mean age at onset	40 years	More common in children than in adults	30 years
Female:male ratio	9:1	Around 1:1	2–4:1
North–South gradient	No increased prevalence with increasing latitude	No data	Increased prevalence with increasing latitude from the equator (either toward North or South)
Prevalence	East Asians: 3.5/100,000 Whites: 1/100,000 Blacks: range from 1.8 to 10/100,000	More common in children than in adults	Up to 100–200/100,000 in White populations, but <5–50/100,000 in many Asian and African countries Rising in most parts of the world
Annual incidence	Around 0.5–0.8/million in Whites Higher annual incidence in non-White populations	Dutch nationwide study: 1.6/million; adults: 1.3/million; children: 3.1/million More data are needed	Up to 100/million in White populations, but was low in many equatorial countries
Disease course	Relapsing	Monophasic or relapsing	Relapsing, with the majority eventually converting to a secondary progressive disease Up to 15% are primary progressive in Whites
Clinical manifestations	Optic neuritis Myelitis Area postrema syndrome Other brain syndromes	Optic neuritis Myelitis ADEM/MDEM Brainstem/cerebral cortical encephalitis Cranial nerve involvement	Optic neuritis Myelitis Brain syndromes
Optic neuritis	Unilateral/chiasmal, long (>1/2 of optic nerve)	Unilateral/simultaneous bilateral, long; frequent optic disc swelling (papillitis)	Unilateral, short
Myelitis	Long (>3 vertebral segments) in 85%; centrally located; affects cervical or thoracic cord	Often long, but may be <3 vertebral segments; gadolinium enhancement less common than AQP4-antibody disease; relatively more common in the lumbosacral region	Non-transverse, short; peripheral/dorsolateral
Attack severity	Moderate to severe	Mild to moderate	Mild to moderate
Recovery	Variable, but commonly poor	Fair to good	Fair to good
Disability	Attack-related	Attack-related	Mainly due to progression
Pathology	Astrocytopathy	Demyelination	Demyelination
Treatment	Immunosuppressants; some MS drugs may be harmful	Consider immunosuppressants if recurrent; some MS drugs may be ineffective	MS disease-modifying drugs

*ADEM/MDEM, acute disseminated encephalomyelitis/multiphasic disseminated encephalomyelitis; AQP4, aquaporin 4; MOG, myelin oligodendrocyte glycoprotein; MS, multiple sclerosis; NMOSD, neuromyelitis optica spectrum disorder*.

## Conclusion

There appears to be varying prevalence rates of NMOSD, most cases of which are AQP4-antibody-positive cases, among the different racial groups worldwide, with East Asians and Blacks having a higher prevalence than Whites. In most regions, these prevalence rates are lower than that of MS. In AQP4-antibody-positive NMOSD, female preponderance is definite (up to 90%) and the majority of the cases are adults. Moreover, the clinical features of NMOSD and disability accrual may be influenced by onset age and race. The data suggest that certain genetic and environmental factors associated with race may be involved in the pathogenesis of NMOSD. More well-designed population-based and longitudinal studies in different geographical areas and racial groups will be useful to clarify the issue, and to shed new lights onto this unique neuroinflammatory disease. Among AQP4-antibody-negative NMOSD, some patients are MOG-antibody-positive, and unlike AQP4-antibody-positive NMOSD, males, and females are equally affected by MOG-antibody-associated disease and the prevalence may be higher in children than in adults. However, the prevalence data of MOG-antibody-associated disease including the ones with an NMOSD phenotype are still insufficient and being accumulated. Accessibility to AQP4-antibody and MOG-antibody testing, which is currently limited in many regions, is a challenge to overcome.

## Author Contributions

JH conceived and designed the study, drafted the manuscript, contributed to data acquisition, and critically revised the manuscript for intellectual content. NA, IN, SB, ML, NK, AJ, RM, BW, FP, SP, JP, DW, JB, and MY made substantial contribution to the intellectual content, contributed to data acquisition, and critically revised the manuscript for intellectual content. KF supervised the study, conceived and designed the study, drafted the manuscript, contributed to data acquisition, and critically revised the manuscript for intellectual content. All authors approved the final manuscript.

## Conflict of Interest

JH, NA, NK, AJ, RM, and JB report no disclosures related to this work. IN has received speaker honoraria and travel funding from Mitsubishi Tanabe Pharma, Biogen Japan, and Novartis Pharmaceuticals, and received research support from LSI Medience Corporation, and has been funded by JSPS KAKENHI Grant Number 17K09772. SB has received honoraria for attendance at advisory boards and travel sponsorship from Bayer-Scherring, Biogen-Idec, Merck-Serono, Novartis, and Sanofi-Genzyme; has received speakers honoraria from Biogen-Idec and Genzyme; is an investigator in clinical trials sponsored by Biogen Idec, Novartis, and Genzyme; and was the recipient of an unencumbered research grant from Biogen-Idec. ML was partly supported by an NHS England highly specialized commissioning group for a neuromyelitis service. She has received support for scientific meetings and honoraria for presentations from Biogen Idec and Novartis and for advisory work from Viela Bio. BW has received royalties from RSR Ltd, Oxford University, Hospices Civil de Lyon, and MVZ Labor PD Dr. Volkmann und Kollegen GbR for a patent of NMO-IgG as a diagnostic test for NMO and related disorders, served on adjudication committee for clinical trials in NMO being conducted by MedImmune and Alexion, and consulted for Chugai, Mitsubishi-Tanabe regarding a clinical trial for NMO. FP has received research support from DFG, BMBF, KKNMS, and the Guthy-Jackson Charitable Foundation. He serves on steering committees of the OCTIMS study (Novartis) and the N-Momentum study (Viela Bio) and has received personal compensation and research support from Alexion, Bayer, Biogen, Roche, Merck, Teva, Shire, Celgene, Novartis, and Sanofi Genzyme. He is an associate editor of Neurology: Neuroimmunology and Neuroinflammation. SP has received grants, personal fees, non-financial support, honorarium, and travel expenses for speaking from Alexion Pharmaceuticals and the Guthy-Jackson Charitable Foundation paid to his institution; grants from Grifols, the National Institutes of Health, and Autoimmune Encephalitis Alliance paid to his institution; consulting fees from Euroimmun paid to his institution; and grants, personal fees, non-financial support, and honorarium from MedImmune; served on the advisory board of Alexion Pharmaceuticals; holds patent 8,889,102 and 9,891,219B2; and has patents pending for GFAP-IgG, Septin-5-IgG, MAP1B-IgG, Kelch-like protein 11, and PDE10A. JP was partly funded by highly specialized services to run a national congenital myasthenia service and a neuromyelitis service. She has received support for scientific meetings and honoraria for advisory work from Merck Serono, Biogen Idec, Novartis, Teva, Chugai, Bayer Schering, Alexion, Roche, Genzyme, MedImmune, EuroImmun, MedDay, Abide ARGENX, UCB, and Viela Bio, and grants from Merck Serono, Novartis, Biogen Idec, Teva, Abide, MedImmune, Bayer Schering, Genzyme, Chugai, and Alexion. She has received grants from the MS society, Guthy-Jackson Charitable Foundation, NIHR, Oxford Health Services Research Committee, EDEN, MRC, GMSI, John Fell, and Myaware for research studies. DW has received research support from Alexion and TerumoBCT and honoraria from MedImmune, Novartis, Biogen, Celgene, Genentech, TG Therapeutics, Arcus Medica, Third Rock Ventures, and Reistone. MY is founder of NovaDigm Therapeutics, Inc, and Metacin, Inc. He is a member of the Genentech Scientific Advisory Committee, and has received travel expenses or honoraria from Genentech and Alexion. KF has received grants from Ministry of Education, Science and Technology of Japan and Ministry of Health, Welfare, and Labor of Japan, and received honoraria, and/or travel expenses for speaking, and/or advisory boards from Mitsubishi Tanabe, Biogen, Bayer, Takeda, Novartis, Alexion, VielaBio, Asahi Kasei, Dainihon Sumitomo, Eisai, Teijin, Ono, Roche, and Chugai.
